# A protection motivation theory-based scale for e-cigarette use assessment among Chinese college students: Development and validation

**DOI:** 10.18332/tid/209411

**Published:** 2025-09-26

**Authors:** Yu Chen, Zining Wang, Jing Xu, Xindou Chen, Yujiang Cai, Si Chen, Kin-Sun Chan

**Affiliations:** 1 School of Art and Communication, Fujian Polytechnic Normal University, Fuqing, China; 2 School of Journalism and Communication, Peking University, Beijing, China; 3 School of Public Health, Peking University, Beijing, China; 4 School of International Studies, Peking University, Beijing, China; 5 China National Center for Food Safety Risk Assessment, Beijing, China; 6 Faculty of Social Sciences, University of Macau, Macau SAR, China

**Keywords:** e-cigarette, protection motivation theory, susceptibility, scale, college students

## Abstract

**INTRODUCTION:**

Electronic cigarettes (e-cigarettes) have gained increasing popularity among young adults worldwide, particularly among college students who represent a key target population for e-cigarette marketing. Understanding cognitive factors that influence e-cigarette use susceptibility is critical for developing effective prevention strategies. This study aimed to develop and validate a scale based on the Protection Motivation Theory (PMT) to assess cognitive factors related to e-cigarette use susceptibility among Chinese college students for prevention purposes.

**METHODS:**

A cross-sectional survey design was employed and data were collected among 303 students aged 18–24 years from universities in China during February 2023. A 21-item PMT scale was adapted from a previous study on Chinese youth tobacco use. Construct validity was assessed using confirmatory factor analysis (CFA). Internal consistency reliability was evaluated using Cronbach's α. Structural equation modeling was used to examine associations between PMT constructs and e-cigarette use susceptibility. Statistical significance was set at p<0.05, and all tests were 2-tailed. Power calculations indicated adequate sample size for the planned analyses. Concurrent validity was examined by correlating PMT constructs with e-cigarette use susceptibility.

**RESULTS:**

After removing one item, the final 20-item scale demonstrated good model fit in the CFA (GFI=0.917, CFI=0.933, RMSEA=0.055). Internal reliability was acceptable to good (Cronbach's α=0.52–0.83). The overall Cronbach's α coefficient was 0.831. All PMT constructs were significantly correlated with e-cigarette use susceptibility in the expected directions (p<0.05).

**CONCLUSIONS:**

The PMT-based scale is a valid and reliable measure to investigate e-cigarette use related cognitions among Chinese college students, and can be used as a tool to guide prevention efforts. The study supports the utility of applying PMT to e-cigarette research in different socio-cultural contexts.

## INTRODUCTION

Electronic cigarettes (e-cigarettes) have been gaining popularity among young adults worldwide, including in China. Recent studies reported e-cigarette use prevalence of 3.8% among college students in Wuhan^1^ and 7.58% among some college students in Zhejiang Province^2^. Compared to the general population, college students are characterized by higher levels of education and health knowledge^3^. Meanwhile, they are a main target of e-cigarette marketing^4,5^. Identifying theory-driven factors that influence e-cigarette use susceptibility in this population is critical for prevention efforts.

Protection Motivation Theory (PMT) provides a useful framework for understanding health-related behaviors^6,7^. It posits that individuals’ motivation to engage in protective behaviors is influenced by threat appraisal (perceived severity and vulnerability) and coping appraisal (perceived self-efficacy, response efficacy, and response costs)^8^. PMT has been applied to investigate conventional cigarette smoking among Western^9^ and Chinese youth populations^10^.

Susceptibility to e-cigarette use, defined as the lack of a firm commitment not to use e-cigarettes in the future, has been identified as a strong predictor of subsequent initiation in youth^11^. Integrating susceptibility with PMT constructs may provide a more comprehensive understanding of cognitive factors influencing e-cigarette use intention and behavior. Assessing factors associated with e-cigarette susceptibility may help identify college students at risk and inform targeted interventions.

As a theoretical guide, PMT has been used in etiological studies to investigate various risk and protective behaviors, including tobacco use^12^, alcohol consumption^7^, physical activity^13^, and HIV prevention^14^. As a conceptual framework, PMT has been utilized in intervention research to develop and evaluate programs for purposeful behavior change^6^.

To our knowledge, no study has utilized PMT to examine e-cigarette use in Chinese college students. To address these gaps, this study aimed to: 1) develop a PMT-based scale assessing e-cigarette use related cognitions among Chinese college students; and 2) evaluate the scale’s reliability, construct validity, and concurrent validity. We hypothesized that the PMT scale would demonstrate acceptable reliability and validity, and that PMT constructs would be significantly associated with e-cigarette use susceptibility.

## METHODS

### Study design, participants and procedures

This study employed a cross-sectional survey design. A convenience sample of 303 college students (150 males, 153 females) aged 18–24 years (mean age=20.6) was recruited from universities in China. Sample size was determined using power analysis for structural equation modeling, with a minimum of 200 participants required for adequate power (>0.80) to detect moderate effect sizes. The inclusion criteria were: 1) current college students; 2) never users of conventional cigarettes and e-cigarettes; and 3) ability to complete an online survey in Chinese. Data were collected in February 2023 using an anonymous online survey. Informed consent was obtained prior to the survey. The study protocol was approved by the Institutional Review Board at Peking University Health Science Center (IRB00001052-23001).

### Measures

*PMT scale*

The 21-item PMT scale was adapted from a previous study on Chinese youth tobacco use^10^. It assessed seven constructs: severity (3 items), vulnerability (3 items), intrinsic rewards (3 items), extrinsic rewards (3 items), self-efficacy (3 items), response efficacy (3 items), and response costs (3 items). All items were rated on a 7-point Likert scale (1= ‘strongly disagree’, 7= ‘strongly agree’).

*E-cigarette use susceptibility*

Susceptibility was measured using three items adapted from previous research^15^: ‘Do you think that you will try an e-cigarette soon?’, ‘If one of your best friends were to offer you an e-cigarette, would you use it?’, and ‘Do you think you will use e-cigarettes in the next 12 months?’. Response options ranged from 1= ‘definitely not’ to 4= ‘definitely yes’. Participants who selected ‘definitely not’ for all three items were classified as not susceptible, and otherwise as susceptible.

### Data analysis

Descriptive statistics were calculated for participant characteristics and scale items. CFA was conducted using maximum likelihood estimation to evaluate the construct validity of the scale. Model fit was assessed using chi-squared/df ratio (<2.0), root mean square error of approximation (RMSEA, ≤0.08), comparative fit index (CFI, ≥0.90), and standardized root mean square residual (SRMR, ≤0.08). Internal consistency reliability was assessed using Cronbach’s α. Structural equation modeling was employed to examine associations between PMT constructs and e-cigarette use susceptibility. Concurrent validity was examined by correlating PMT construct scores with e-cigarette use susceptibility using Spearman correlations given the non-normal distribution of some variables and the relatively small sample size. Statistical significance was set at p<0.05, and all statistical tests were two-tailed. All analyses were conducted using SPSS 27.0 and AMOS 27.0.

## RESULTS

### Sample characteristics

Among the 303 participants, 150 (49.5%) were male and 270 (89.11%) were undergraduate students. The average age was 20.6 years (SD=1.67) (Table 1); 18.81% were susceptible to e-cigarette use.

**Table 1:** Participant characteristics, a cross-sectional survey, Universities in China, February 2023 (N=303)

*Characteristics*	*Male* *n (%)*	*Female* *n (%)*	*Total* *n (%)*
**Total**	150 (49.50)	153 (50.50)	303 (100)
**Age** (years), mean (SD)	20.59 (2.04)	20.63 (2.20)	20.6 (1.67)
**Education level**			
Three-year college	5 (3.33)	6 (3.92)	11 (3.63)
Undergraduate	133 (88.67)	137 (89.54)	270 (89.11)
Master’s degree student	12 (8.00)	9 (5.88)	21 (6.93)
PhD student	0 (0.00)	1 (0.65)	1 (0.33)

### Item analysis and reliability

The mean scores of the 21 PMT items ranged from 1.65 to 6.59 (Table 2). Item 18 was removed due to non-significant item-total correlation. The final 20-item scale had a Cronbach’s α of 0.831, indicating good overall reliability. The Cronbach’s α for the subscales ranged from 0.52 (response efficacy) to 0.83 (intrinsic rewards). Although the response efficacy subscale had a relatively low α of 0.52, it was considered acceptable given it only contained two items.

**Table 2:** Item responsiveness and reliability analysis of the PMT scale after deletion of Q18, a cross-sectional survey, Universities in China, February 2023 (N=303)

*Factor/item*	*Mean (SD)*	*Overall* *relevance*	*Cronbach’s α*
F1 Severity	6.01 (0.87)		0.75
Q1 The longer you vape, the more harmful they become	6.47 (0.72)	0.480^b^	0.82
Q2 Vapers are more likely to get sick than non-vapers	5.98 (1.11)	0.516^b^	0.82
Q3 Vapers more likely to die earlier than non-vapers	5.57 (1.29)	0.588^b^	0.82
F2 Vulnerability	5.32 (1.25)		0.78
Q4 If I vape, I am likely to become addicted to them	5.17 (1.70)	0.357^b^	0.84
Q5 If I vape, I may get sick from them	5.52 (1.30)	0.621^b^	0.82
Q6 If I vape, I’m likely to die early	5.28 (1.49)	0.591^b^	0.82
F3 Intrinsic rewards	3.24 (1.24)		0.79
Q7 Vaping can make people comfortable	3.32 (1.61)	0.575^b^	0.82
Q8 Vaping can lead to energy concentration	3.09 (1.46)	0.563^b^	0.82
Q9 Vaping helps you think	2.88 (1.38)	0.562^b^	0.82
F4 Extrinsic rewards	3.86 (0.93)		0.63
Q10 Vaping is cool and trendy	1.84 (1.16)	0.496^b^	0.82
Q11 Vaping can help with socialization	2.56 (1.47)	0.514^b^	0.82
Q12 Vapers are likely to be happier in their lives than non-vapers	2.40 (1.29)	0.525^b^	0.82
F5 Self-efficacy	6.46 (0.79)		0.74
Q13 If I don’t want to vape, no one can convince me to	6.35 (1.17)	0.363^b^	0.83
Q14 Even if everyone around me becomes a vaper, it doesn’t mean I have to vape	6.46 (0.97)	0.357^b^	0.83
Q15 Even if a relative or friend asks me to vape, I can refuse to	6.59 (0.73)	0.390^b^	0.83
F6 Response efficacy	5.86 (1.00)		0.52
Q16 Feeling good without vaping	6.17 (1.12)	0.528^b^	0.82
Q17 People will be less likely to get sick if they don’t vape	5.56 (1.31)	0.403^b^	0.82
F7 Response costs	4.16 (0.79)		0.67
Q19 Not vaping can make it difficult to fit in with some friends	2.28 (1.38)	0.415^b^	0.83
Q20 It’s rude to refuse someone’s e-cigarette	1.96 (1.06)	0.441^b^	0.83
Q21 Not vaping takes away an opportunity for enjoyment	1.65 (0.90)	0.560^b^	0.82

a Significant correlation at p<0.05.

b Significant correlation at p<0.01.

### Construct validity

The initial CFA yielded inadequate model fit: chi-squared/df=3.12, CFI=0.85, RMSEA=0.08, SRMR=0.07. After allowing error covariances between three pairs of items based on modification indices and theoretical considerations, the final CFA demonstrated good model fit: chi-squared/df=1.92, CFI=0.93, RMSEA=0.06, SRMR=0.05. All factor loadings were significant at p<0.001 (Table 3).

**Table 3:** Correlation of protection motivation theory components with e-cigarette use susceptibility by sex, a cross-sectional survey, Universities in China, February 2023 (N=303)

*Dimension*	*Male*		*Female*		*Total*	
	*r*	*p*	*r*	*p*	*r*	*p*
**TA**	0.313	<0.01	0.267	<0.01	0.265	<0.01
PT	-0.259	<0.05	-0.167	<0.05	-0.185	<0.01
S	-0.296	<0.01	-0.211	<0.01	-0.229	<0.01
V	-0.184	0.107	-0.098	0.144	-0.113	<0.05
PR	0.204	0.073	0.258	<0.01	0.23	<0.01
IR	0.149	0.192	0.229	<0.01	0.2	<0.01
ER	0.203	0.075	0.217	<0.01	0.199	<0.01
**CA**	-0.285	<0.05	-0.316	<0.01	-0.302	<0.01
PE	-0.32	<0.01	-0.305	<0.01	-0.307	<0.01
RE	-0.278	<0.05	-0.2	<0.01	-0.212	<0.01
SE	-0.232	<0.05	-0.3	<0.01	-0.284	<0.01
RC	0.112	0.327	0.187	<0.01	0.157	<0.01

TA: threat assessment. PT: perceived threat. S: severity. V: vulnerability. PR: perceived reward. IR: intrinsic reward. ER: extrinsic reward. CA: coping assessment. PE: perceived efficacy. SE: self-efficacy. RE: response efficacy. and RC: response cost.

### Concurrent validity

As shown in Table 3, all PMT constructs, perceptions, and pathways were significantly correlated with e-cigarette use susceptibility in the expected directions. Perceived severity (r= -0.23, p<0.01), vulnerability (r= -0.11, p<0.05), and threat appraisal (r= -0.19, p<0.01) were negatively correlated with susceptibility. Intrinsic rewards (r=0.20, p<0.01), extrinsic rewards (r=0.20, p<0.01), perceived rewards (r=0.23, p<0.01), and coping appraisal (r=0.27, p<0.01) were positively correlated with susceptibility. Self-efficacy (r= -0.28, p<0.01), response efficacy (r= -0.21, p<0.01), and perceived efficacy (r= -0.31, p<0.01) were negatively correlated with susceptibility, while response costs (r=0.16, p<0.01) were positively correlated.

The structural equation model examining associations between PMT constructs and e-cigarette use susceptibility demonstrated good model fit: chi-squared/df=1.97, CFI=0.92, RMSEA=0.06, SRMR=0.06. Severity (β= -0.22, p=0.002), intrinsic rewards (β=0.29, p<0.001), self-efficacy (β= -0.31, p<0.001) and response costs (β=0.18, p=0.03) were significantly associated with susceptibility (Table 3). The model accounted for 27.3% of the variance in susceptibility.

## DISCUSSION

This study developed and validated a 20-item PMT scale for assessing e-cigarette use related cognitions among Chinese college students. The scale demonstrated acceptable to good reliability and validity. The CFA confirmed the seven-factor structure of the scale. All PMT components significantly correlated with e-cigarette use susceptibility in the expected directions, supporting the concurrent validity of the scale. These findings are consistent with PMT and previous research on tobacco use^12,16^. The scale provides an important measurement tool for conducting theory-driven research on e-cigarette use in China.

We found that perceived threat, severity, and vulnerability were negatively associated with e-cigarette susceptibility; while coping appraisal, perceived rewards, intrinsic rewards, and extrinsic rewards were positively associated with susceptibility. Self-efficacy, response efficacy, and perceived efficacy were negatively associated with susceptibility, while response costs were positively associated. These patterns align with PMT hypotheses^16,17^. The results suggest that individuals with lower e-cigarette susceptibility perceive higher e-cigarette related threats and lower rewards, while those with higher perceived rewards, lower coping efficacy, and higher coping costs are more susceptible to e-cigarette use. These findings deepen our understanding of the social cognitive processes influencing Chinese youth’s e-cigarette use intention and behavior, providing a basis for developing targeted interventions.

Previous research has shown similar patterns using PMT to understand tobacco-related behaviors among adolescents and young adults^16,18^, indicating that threat appraisal and coping appraisal constructs were significant predictors of e-cigarette use intentions and behaviors. Our findings extend this research to Chinese college students, demonstrating the cross-cultural applicability of PMT in understanding e-cigarette-related cognitions.

To our knowledge, this is the first study to adapt and validate a PMT scale for e-cigarette use in Chinese college students. The scale can serve as a useful tool for exploring cognitive determinants of e-cigarette use and identifying high-risk individuals in this population. The assessment of PMT cognitions may help guide the development of targeted health communication messages and intervention strategies, such as increasing awareness of e-cigarette harms, reducing perceived rewards, enhancing self-efficacy to refuse e-cigarettes, and emphasizing the costs of e-cigarette use.

### Limitations

The results should be interpreted with caution due to several limitations. First, the cross-sectional design precludes causal inferences between PMT constructs and e-cigarette susceptibility. Longitudinal studies are needed to establish the temporal relationships between PMT perceptions and e-cigarette initiation. Second, convenience sampling was used, which may limit the generalizability of findings to the broader population of Chinese college students. The use of convenience sampling may introduce selection bias, as participants may differ systematically from nonparticipants in unmeasured characteristics. Future research should validate the scale in more diverse populations and settings using probability sampling methods. Third, the relatively small sample size (n=303) may limit the stability of factor loadings and correlation estimates. Fourth, the response efficacy subscale demonstrated low internal consistency reliability (Cronbach’s α=0.52), which may affect the validity of findings related to this construct. Fifth, potential residual confounding from unmeasured variables cannot be ruled out in this observational study. Sixth, e-cigarette use behavior was not assessed in this study. Future research should examine the utility of the PMT scale in prospectively assessing actual e-cigarette use. Finally, the measures relied on self-report and may be subject to recall and social desirability bias.

## CONCLUSIONS

This study suggests that PMT is a relevant theoretical framework for understanding e-cigarette use among Chinese college students. The 20-item PMT scale demonstrated satisfactory reliability and validity. It can be used for identifying students susceptible to e-cigarette use and developing targeted prevention interventions. The findings provide empirical support for applying behavioral theories to e-cigarette research in different socio-cultural contexts. Overall, this study provides an important assessment tool for conducting theory-driven research and interventions on e-cigarette use in China.

## Data Availability

The data supporting this research are available from the authors on reasonable request.
